# The Future is The Past: Methylation QTLs in Schizophrenia

**DOI:** 10.3390/genes7120104

**Published:** 2016-11-24

**Authors:** Anke Hoffmann, Michael Ziller, Dietmar Spengler

**Affiliations:** Max Planck Institute of Psychiatry, Translational Psychiatry, Munich, 80805, Germany; hoffmann@psych.mpg.de (A.H.); michael_ziller@psych.mpg.de (M.Z.)

**Keywords:** schizophrenia, methylation quantitative trait loci, fetal brain, genome-wide association studies, non-coding variants, induced pluripotent stem cells, DNA memory

## Abstract

Genome-wide association studies (GWAS) have remarkably advanced insight into the genetic basis of schizophrenia (SCZ). Still, most of the functional variance in disease risk remains unexplained. Hence, there is a growing need to map genetic variability-to-genes-to-functions for understanding the pathophysiology of SCZ and the development of better treatments. Genetic variation can regulate various cellular functions including DNA methylation, an epigenetic mark with important roles in transcription and the mediation of environmental influences. Methylation quantitative trait loci (meQTLs) are derived by mapping levels of DNA methylation in genetically different, genotyped individuals and define loci at which DNA methylation is influenced by genetic variation. Recent evidence points to an abundance of meQTLs in brain tissues whose functional contributions to development and mental diseases are still poorly understood. Interestingly, fetal meQTLs reside in regulatory domains affecting methylome reconfiguration during early brain development and are enriched in loci identified by GWAS for SCZ. Moreover, fetal meQTLs are preserved in the adult brain and could trace early epigenomic deregulation during vulnerable periods. Overall, these findings highlight the role of fetal meQTLs in the genetic risk for and in the possible neurodevelopmental origin of SCZ.

## 1. Introduction

Schizophrenia (SCZ) is a chronic, debilitating disease characterized by the presence of positive, negative, and cognitive symptoms that affect multiple aspects of mental activity, including perception, thought, attention, memory, and emotion. The age of onset is typically adolescence or early adulthood, with a median lifetime prevalence of 4.0 per 1000 and a morbid risk of 7.2 per 1000 [1].

In this review, we will firstly discuss the current status of SCZ genetics and the urgent need to map genetic variation-to-genes-to-function. Recent progress on genome-wide functional annotation of DNA sequences opens up the perspective to prioritize genetic variation to define causal variants. Here, we will explore new insights into the role of dynamic DNA methylation and ask how genetic influences on DNA methylation could contribute to the molecular etiology of SCZ. Next, we will analyze current evidence for the presence of methylation quantitative trait loci (meQTLs) in peripheral and, particularly, in healthy and diseased brain tissues, and their potential role in transcription and RNA splicing. Most interestingly, we will discuss recent findings on the role of CpG methylation and meQTLs in fetal brains and how this integrated knowledge could inform about epigenomic deregulation during vulnerable periods of brain development and the early origin of SCZ.

## 2. The Genetic Architecture of SCZ

Some 40 years of epidemiological and genetic studies have shown that SCZ is a complex disorder of combined genetic and environmental causation [2]. Family history is a strong and widely replicated risk factor, and the heritability of SCZ exceeds 60% in two national family studies [3,4] and 80% in twin studies [5]. At the same time, increasing evidence has accumulated for an important role of environmental risk factors in SCZ including aversive perinatal and early childhood events (e.g., maternal stress or illness, infection, severe socioeconomic disparity), belonging to an immigrant group or growing up in an urbanized area [3,4,5,6].

A broad spectrum of approaches (genetic epidemiology, segregation analysis, cytogenetics, genome-wide linkage, candidate gene associations, genome-wide association studies (GWAS), copy number variants (CNVs), and resequencing) have been applied to elucidate the genetics of SCZ [7]. Overall, these data support a role for both common and rare risk variants in SCZ, which occur with varying degrees of frequency and confer different risks for disease development. The polygenicity of SCZ is diverse and involves many common variants of subtle effects, rare but highly penetrant CNVs, and possibly exome variants [2]. In any case, loci identified so far represent probably only a fraction of the existing variants. The authors of a recently published GWAS [8] hypothesized that approximately 6300–10,200 independent and mostly common single nucleotide polymorphisms (SNPs) could underlie the risk for SCZ, an estimate that agrees well with the prediction that over 12,000 SNPs confer an effect on SCZ and bipolar disorder (BIP) [9]. Although each single SNP confers only a tiny increase in risk, together, they incrementally account for around 50% of the total variance in liability to SCZ [8]. In view of SCZ heritability around 60% [3,4], these estimates indicate that common genetic variation underlies major parts of SCZ heritability and emphasize the ongoing need for adequately powered studies.

Still, each genetic finding can provide a potential etiological clue and point to a discrete number of biological and developmental pathways whose dysfunction typifies SCZ. Dissection of numerous relevant loci at the network scale [10] might help to unify the polygenic nature of SCZ and offer new opportunities for therapeutic interventions [11].

## 3. The GWAS Era

The advent of high-throughput genotyping technologies has provided important insights into the genetic architecture of SCZ, bipolar disorder (BIP), and major depression (MDD) [2]. The HapMap and 1000 Genome projects originally identified ≈40 million genetic variants across the human genome consisting of insertion/deletions (indels), CNVs, inversions, and SNPs. The most frequent form of genetic variation are SNPs, which account for 95% of all known sequence variants [12]. Up to now more than 85 million SNPs have been identified in the human population [13].

Array systems for GWAS are designed to incorporate only tag SNPs that represent all SNPs in the same linkage disequilibrium (LD) block. These tag SNPs capture most human genome variation through haplotype-based SNP imputation [14,15]. SNPs that are statistically over-represented in disease populations are termed risk-associated SNPs, whereby multiple associations are likely to all tag a single causal variant. Since independently-associated SNPs do not refer to well-bounded chromosomal regions, it is convenient to define physical boundaries for the found SNP associations to identify candidate risk genes. For example, a recent GWAS on SCZ [16] detected 128 independent associations and defined an associated locus as the physical region comprising all SNPs correlated at *r*^2^ > 0.6 with each of the 128 tag SNPs. Further, associated loci within 250 kb of each other were merged and resulted in 108 physically distinct associated loci, to which we refer in the remainder of this review as Psychiatric Genomics Consortium (PGC) risk variants. Among these, a notable number of 83 risk loci have not been implicated previously in SCZ.

In contrast to Mendelian diseases that are caused by mutations in the coding region of a gene, risk-associated SNPs frequently map to non-coding genomic regions equally represented by intergenic and intronic regions [17]. Importantly, tag SNPs capture all the other SNPs present at the risk-associated haplotype block, but are, themselves, not necessarily the causal genetic variants that underlie the association. LD calculation [18,19,20] together with the 1000 Genome Project reference panels from different populations [12] has been used to include SNPs unrelated to the tag SNPs contained on the standard GWAS arrays. Alternatively, fine-mapping studies applied dense genotyping arrays that include all common SNPs within the previously defined risk loci. Still, not only does the vast majority (≈93%) of cataloged tag SNPs map to non-coding regions, but also most SNPs in high LD with the risk-associated tag SNPs and most SNPs identified by fine-mapping.

## 4. In Search of Function

GWAS detect broad genomic loci containing multiple, sometimes hundreds, of correlated disease-associated variants without defining the gene(s) actually underlying the disease. These results raise three important questions: (1) which variant(s) contribute to the disease association; (2) through which genes/transcripts do these variants act; and (3) what is the molecular mechanism of action that relates to the disease phenotype.

To translate the wealth of genetic findings into transformative insight into disease processes and possible therapies, it is necessary to map genetic variation-to-genes-to-function. Population-based studies have shown that genetic variants can modulate gene expression [21,22], transcription factor (TF) binding [23], chromatin accessibility as assessed by DNase I hypersensitivity [24], histone modifications [23,25,26], DNA methylation [27], and RNA splicing [28]. Moreover, genetic variations associate more often with a particular phenotype if they localize within DNase I hypersensitive regions or differentially methylated regions from a disease-relevant cell type [29].

Until recently, the functional characterization of risk-associated loci has been hampered by the insufficient annotation of non-coding sequences. A series of large-scale genomics projects, comprising Encyclopedia of DNA Elements (ENCODE), Roadmap Epigenomics, International Human Epigenome Consortium (IHEC), and Functional Annotation of the Mammalian Genome (FANTOM) has addressed these limitations by taking advantage from recent advances in massively parallel sequencing-based technologies to produce genome-wide maps of functional elements of the non-coding regions in different cell and tissue types across distinct developmental stages [30]. The result is a comprehensive landscape of epigenomic elements regulating gene expression via DNA methylation, histone modifications, and non-coding RNAs (ncRNAs). Many of these functional annotations can serve to inform on a regulatory role of disease-associated genetic variations. Here, we will focus on DNA methylation and consider the influence of genetic variation on the brain methylome. Following, we will discuss exciting new findings on the impact of risk variants from GWAS for DNA methylation and their potential roles in the origin, manifestation, and course of SCZ.

## 5. Dynamic DNA Methylation

Insight into the nature of molecular epigenetic mechanisms has greatly benefited from the elucidation of the role of DNA methylation, posttranslational modifications of core histones, nucleosome positioning, and ncRNAs [31].

In humans, canonical DNA methylation refers to the transfer of a methyl group to cytosine residues at the carbon 5 position within the dinucleotide CpG. This process is catalyzed by DNA methyltransferases (DNMTs), a family of enzymes responsible for de novo (DNMT1) and maintenance (DNMT3A and DNMT3B) DNA methylation. In the genome, CpG sites are in general depleted and clustered in regions termed CpG islands (CGIs). In vertebrates, these regions predominantly locate at gene promoters (except humans where less than half of the CGIs locate at promoters) and stay methylation-free [32,33]. Oppositely, DNA methylation of promoter CGIs commonly associates with a closed chromatin state inaccessible to regulatory transcription complexes and with gene silencing [34,35]. In general, however, inactive CGI do not acquire DNA methylation but recruit Polycomb complexes that catalyze methylation at lysine 27 of histone H3 (H3K27me3), a modification causing chromatin condensation and gene silencing [36,37].

Similarly, the popular view of DNA methylation as an all-purpose repressive mark needs to be revised in light of recent findings that gene body methylation is also common in ubiquitously expressed genes where it promotes prolongation efficiency and alternative promoter usage, suppresses spurious initiation of transcription, and positively correlates with gene expression [32]. In fact, genome-wide methylation analyses show that the effects of DNA methylation depend on the primary sequence, genomic location, and pre-existing transcriptional activity contributing together to both gene activation and repression.

More recently, the transformative discovery of active demethylation via iterative oxidation catalyzed by the family of ten–eleven translocation enzymes has assigned to DNA methylation a more dynamic role in gene regulation than originally thought [38]. These insights suggest that remodeling of the DNA methylation landscape over the course of development occurs in a temporally and spatially highly constrained manner, affecting small regions of the genome harboring regulatory function.

In light of these findings, dynamic DNA methylation has also been implicated in the long-term impact of the environment on post-mitotic neuronal cells. Neuronal signaling can couple to the epigenetic machinery or, more commonly, to transcriptional regulators that in turn recruit the epigenetic machinery. Neuronal activity-dependent TFs are known to direct the chromatin-DNA response to specific target genes at their DNA-address code and can lead to long-lasting marks that persist beyond the initial stimulus. In short, dynamic DNA methylation provides an intriguing mechanism by which long-term environmental effects are encoded in the genome [39].

## 6. What Are meQTLs?

In 2001, Jansen and Nap firstly introduced the term “genetical genomics” to describe an approach for the identification of genes regulated by genetic variation [40]. meQTLs and expression quantitative trait loci (eQTLs) are similar to other QTLs that can affect any given trait of interest (e.g., body weight, growth rate, and disease risk) and are identified by measuring DNA methylation or gene expression, respectively, in panels of genetically different, genotyped individuals. To infer QTLs, statistical association tests are applied to compare methylation or expression levels with the respective genotype of each individual.

Accordingly, meQTLs are genomic regions that contain one or more DNA sequence variants that influence the methylation level (typically CpG methylation) of other DNA regions that can contain a given regulatory region(s), genic region(s), or region(s) of unknown function. Relatedly, eQTLs contain genetic variations that influence the expression level (typically mRNA abundance) of a given gene(s) [41].

**Figure 1:**
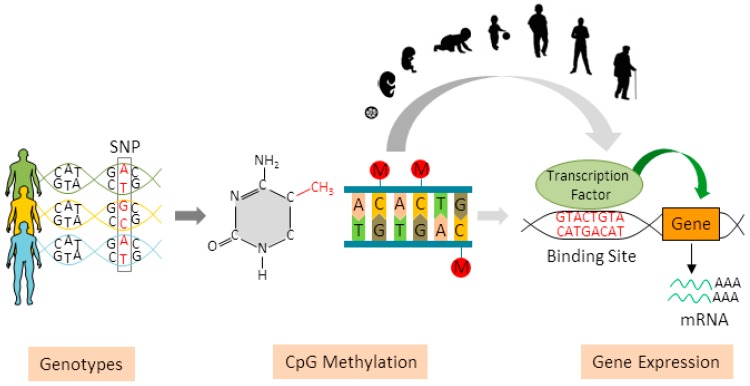
Role of methylation quantitative trait loci (meQTLs) for gene expression. Genetic variation influences levels of DNA methylation at regulatory regions and can modulate gene expression leading to decreased or increased gene transcription. Importantly, the effects of genetically induced changes in DNA methylation critically depend on developmental stage, and possibly, environmental context. SNP: single nucleotide polymorphism.

It is important to note that these genetically induced changes in DNA methylation can, but do not necessarily have to result in changes of gene expression. Possible explanations for this phenomenon include regulatory events that do not affect overall expression levels (e.g., RNA splicing), temporal-spatial constraints (e.g., meQTL regulating transcription during specific developmental stages) or context dependency (e.g., meQTL regulating activity-dependent transcription) (Figure 1).

It is also important to note that results on meQTLs have to be distinguished from those on allele-specific changes in DNA methylation that can help to maximize the information content from GWAS [42]. Such allele-specific differences in DNA methylation can also act in the mediation of gene–environment interactions. For instance, allele-specific methylation at an intronic regulatory site of the cochaperone *FKBP5* in response to early life adversity is thought to confer risk for later stress-related disorders [43]. These and related findings [44,45] do not match, however, the criteria of a meQTL and are beyond the scope of the present review.

meQTLs and eQTLs are commonly distinguished according to the relative locations of the QTL and the gene(s) that they affect, and by the mechanism through which they influence DNA methylation or gene expression. In the following part we will focus on meQTLs in the knowledge that most of the afore-noted will also apply to eQTLs and will refer to the latter when necessary.

### 6.1. Local meQTLs

Local meQTLs reside nearby the gene(s) they influence and are common in human peripheral tissues [46,47,48,49] and brain [50,51,52] (see further below). Historically, investigations on local eQTLs preceded the ones regarding meQTLs due to the obvious link to gene activity and the easiness of RNA measurements.

**Figure 2:**
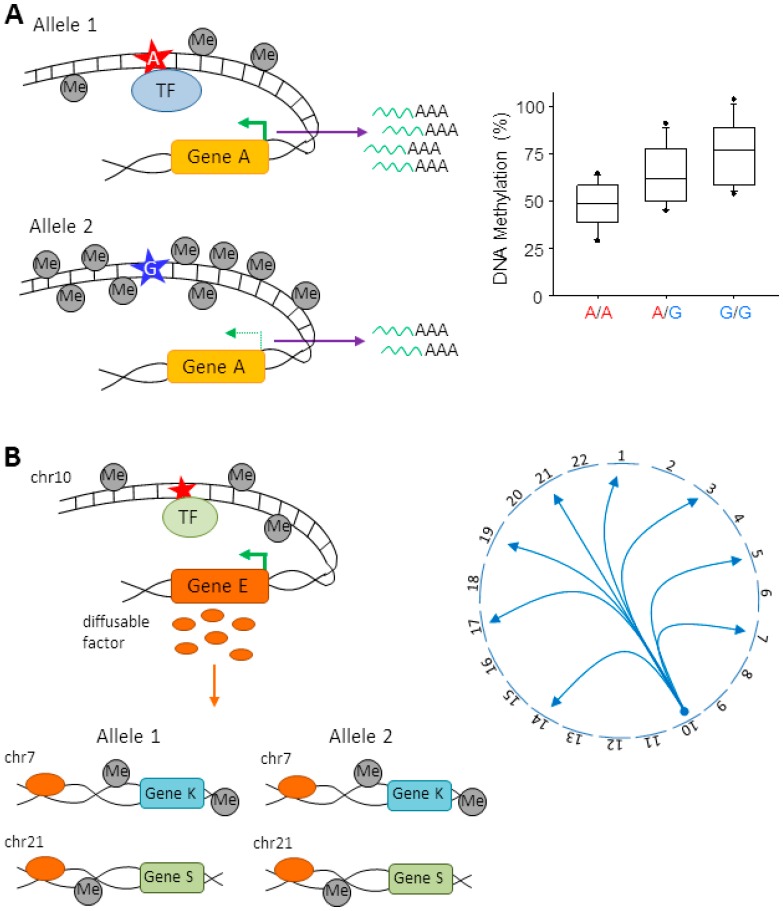
Model for local meQTLs: (**A**) A local *cis*-acting variant (A-allele symbolized by a red star) resides in a regulatory element, for example a transcription factor (TF) binding site. Sequence variation (G-allele symbolized by a blue star) can result in reduced binding of the TF, diminished transcription of gene A, and encroachment of DNA methylation (**left**). As depicted by the chart, *cis*-meQTLs show differences in the amount of CpG-methylation (Me) between the two copies of an allele. Homozygous carriers of the transcriptionally active A-allele show less DNA methylation when compared to homozygous carriers of the transcriptionally less active G-allele or heterozygous carriers (**right**); (**B**) *Trans*-meQTLs result from differences in the expression, structure, or function of a diffusible factor that is equally available to both alleles at target sites. Accordingly, target sites (genes K and S) do not show differences in allele-specific methylation rates. *Trans*-meQTLs can involve variation in the sequence of TF binding sites (red star) driving expression of the diffusible factor or variation in the coding sequence of the diffusible factor leading to altered structure or function (**left**). Functionally, *trans*-meQTLs can affect transcription levels of multiple genes. Such *trans* associations can be shown on a circle plot (chromosomes (chr) labeled 1–22 with arrows pointing to location of a gene on a given chromosome) (**right**).

Local meQTLs presumably regulate DNA methylation via two different routes. Firstly, they can act in *cis* and influence DNA methylation in an allele-specific manner. Hereby, the allele encoding the *cis*-meQTLs influences only the methylation of the copy of the gene that localizes on the same physical chromosome but not the methylation of the gene copy on the homologous chromosome (Figure 2A). Quantification of the relative DNA methylation levels of the two alleles enables the identification of *cis*-meQTLs in heterozygous individuals. Many studies agree that *cis*-acting QTLs have large effects size that can be detected in less than 100 samples [24,46,53,54].

Secondly, local meQTLs can act in *trans* through polymorphisms that alter the expression, structure or function of a diffusible factor [55]. The subsequent differential abundance or activity of this factor influences expression levels of the gene(s) that are regulated by the *trans*-meQTL (Figure 2B). Since the diffusible factor is equally available to both alleles, *trans*-meQTLs lack an allelic bias in DNA methylation in heterozygous individuals.

### 6.2. Distant meQTLs

Distant meQTLs correspond to loci that map further away from the gene(s) they regulate. The term distance refers to genetic distance and varies depending on the organism, ranging from several kilobases in yeast to several megabases in human. Traditionally, distant meQTLs have been thought to act in *trans.* This view needs, however, to be revised in light of recent findings on the highly dynamic and hierarchically structured nuclear architecture [56]. As a result, distant meQTLs can physically contact target genes through three-dimensional looping and mimic the effects of *cis*-meQTL.

Distant meQTLs and eQTLs have been harder to detect in human populations than in experimental crosses of model organisms. Human population samples contain multiple haplotypes at most positions in the genome, multiple variants per region, and shorter linkage blocks that altogether require a much higher number of association tests. Additionally, they show also smaller effect sizes and appear to be more tissue-specific than local meQTLs and eQTLs (see further below).

## 7. Molecular Mechanisms for meQTLs

*Cis*-acting QTLs typically entail allele-specific differences in regulatory DNA elements. For example, SNPs in the DNA-address code of TFs can influence DNA binding to the *cis*-allele, followed by altered transcription, and encroachment of methylation. DNA-binding of the TF could be unaltered by DNA methylation at its recognition site or neighboring sequences but merely prevent the encroachment of methylation [57,58]. Alternatively, methylation-sensitive TF binding could enforce the effects of genetic variation at the DNA-address code by negative feedback. Well-fitting this hypothesis, TF binding sites are enriched in differentially methylated regions (DMR) between individual and cell types [27] and germ layer-dependent rewiring of TF binding sites is likely to drive changes in CpG methylation [59]. *Cis*-meQTLs related to these events do not only vary in TF binding itself but also in differential histone modifications, DNase I and chromatin accessibility, and mRNA levels [60] (see further below).

Moreover, allele-specific differences in SNPs that create or disrupt CpGs (“CpG SNPs”) can influence the propensity of a TF-binding site or neighboring non-polymorphic CpGs to undergo methylation and hereby favor or disfavor TF-binding and/or a closed chromatin structure. Lastly, recent reports suggest a direct role of sequence variation on site- and region-specific DNA methylation with DNA sequence itself having an important role in the maintenance of DNA methylation [57,61].

*Trans*-meQTLs can result from coding variants in genes encoding diffusible regulatory factors or local meQTLs of such genes. TFs, signaling pathways, modifiers of DNA or chromatin are likely candidates for *trans*-meQTLs. Since few *trans*-meQTLs have been fine-mapped so far (see above), the molecular causes of *trans*-acting variation in DNA methylation are yet incompletely understood.

## 8. Early meQTL Studies in the Brain

A number of studies have shown that genetic variation frequently associates with quantitative differences in methylation levels in human cell lines [46,60], peripheral tissues [48,62], and the brain [50,63].

Early studies on brain methylation involved low-resolution CpG profiling of promoter-biased regions (i.e., the Illumina HumanMethylation27 Bead Chip array containing 27,578 individual CpG sites spanning 14,495 genes). In 2010, Zhang and coworkers genotyped DNA from 153 samples from adult cerebellum from patients with various psychiatric diseases and normal controls of European ancestry and mapped interindividual differences in DNA methylation at 8590 CpG sites to 6229 genes [63]. Differences in CpG methylation were most enriched at CGI shores that often contain tissue-specific methylation sites [64] and at further distances from CGIs. Among the differentially methylated CpGs, 736 CpG sites significantly associated in *cis* with 2878 SNPs, whereby this effect was stronger for CGIs versus non-CGI regions. Additionally, 12 CpG sites associated in *trans* with 38 SNPs. DNA methylation influenced gene expression in a subgroup of 85 genes associated with a meQTL and in most of these cases DNA methylation negatively correlated with gene expression. Moreover, 10 genes among this subgroup showed for the same SNP a significant association with both DNA methylation and gene expression with DNA methylation significantly correlating with gene expression.

Using the same platform, Gibbs and co-investigators [50] identified in four brain regions (caudal pons, cerebellum, frontal and temporal cortex) from 150 normal Caucasian individuals an abundance of *cis*-meQTLs and *cis*-eQTLs. In accord with previous findings [21,65,66], the majority of large effect eQTLs was preserved across different tissues, whereas many small eQTLs were tissue-specific. The researchers also detected a large number of meQTLS (>5000 in cerebellum, frontal and temporal cortex) with a substantial conservation across tissues (>2800). Most of the meQTLs showed effect sizes similar to eQTLs and localized predominantly in *cis*. In agreement with the results from above, few meQTLs were found to statistically associate with DNA methylation and mRNA expression levels.

While these early studies suggest an abundance of meQTLs in different human brain tissues that largely reside in *cis* at upstream regulatory sites, it is important to remember that distant meQTLs are harder to detect in human populations (see section 6.2). Irrespective of this reservation, meQTLs are of similar effect size as eQTLs and correlations with gene expression are few and can be both positive and negative.

## 9. meQTLs Are Enriched at Regulatory Sites

A recent study [67] comprehensively analyzed genetic and epigenetic influences on genome regulation across different cell types and identified over 20,000 meQTLs. Previously, the researchers had genotyped 2.5 million SNPs, assessed methylation levels of 482,421 CpGs (Illumina Infinium HumanMethylation450 BeadChip) and mRNA-sequenced the transcriptomes from fibroblasts, T cells, and lymphoblastoid cell lines (LCLs) derived from 204 umbilical cords of newborn children [63]. In agreement with previous studies [24,30], eQTLs were enriched in DNase I hypersensitive sites at CGI-shores, gene bodies and enhancers, and consistently affected gene expression across cell types. In contrast, meQTLs were enriched in enhancers and insulators and localized distant to the transcription start site. This result resembles previous findings on the location of DMRs between differentiating cell types [27] and raises the possibility that the same methylation sites involved in tissue differentiation also contribute to inter-individual variability determined by genetic variation [62,67]. meQTLs significantly associated with gene expression (fibroblasts ≈4%, T-cells ≈11%, and LCLs ≈16%) and influenced gene expression both positively and negatively (negative associations were ≈5% in fibroblasts, ≈69% in T-cells, and ≈57% in LCLs). In most cell types, combined meQTL preferentially mapped to CGI shores, gene bodies, and enhancers whereby the effects on gene expression were more tissue-specific compared to eQTLs.

Functionally, DNA methylation associated with TF abundance and Spearman’s rank correlation between methylation levels (within 50 kb on either side of the transcription start sites) and alternative splicing levels revealed for many of the genes tested a significant association that was cell type-specific.

Briefly, this study corroborates previous findings on the preferential localization of meQTLs at upstream regulatory sites and points to a complex relationship between associated DNA methylation and gene expression. Further, genomic and epigenomic variations can contribute to alternative splicing as well as to the tissue specificity of some of these interactions.

In light of these studies [50,63,67], the question remains whether the detected methylation changes are a cause or consequence of the gene expression changes.

To approach this issue, Banovich and co-investigators [60] comprehensively investigated correlations between genetic variation, DNA methylation, RNA expression, DNase I hypersensitive sites, different histone modifications (H3K4me1, H3K4me3, H3K27ac, H3K27me3), and polymerase II binding in 64 well-characterized HapMap Yoruba LCLs. Genetic variation associated with methylation levels at nearly 14,000 CpG sites. Interestingly, SNPs disrupting TF binding sites were more likely to be associated with DNA methylation levels than SNPs localizing within DNase I hypersensitive sites but not in TF binding sites. This result suggests that changes in TF binding frequently trigger a regulatory cascade that drives concerted changes in multiple epigenetic mechanisms and, ultimately, transcription. In accordance with recent work of Gutierrez-Arcelus and co-investigators [67], meQTLs were under-represented at promoters but enriched at distant regulatory elements consisting of enhancers and insulators. SNPs that concurrently associated with DNA methylation and gene expression often showed a positive correlation between DNA methylation and gene expression. This result corroborates previous findings that DNA methylation at distant regulatory sites can have an activating effect on gene transcription [46,50,62]. A plausible explanation of these findings is that methylation of upstream regulatory sites could block methylation-sensitive DNA binding of repressor proteins such as CCCTC-binding factor (CTCF) or RE1-silencing transcription factor (REST) [57,58].

Overall, these findings indicate that meQTLs are more likely to reside at distant regulatory elements than at promoters [50,60,63,67] and coincide with TF binding, chromatin conformation, gene expression, RNA splicing, and potentially, disease risk [29].

## 10. meQTLs in SCZ

The first study on meQTLs in psychiatric diseases did not investigate SCZ, but BIP [68]. Since BIP shares a substantial fraction of the genetic risk with SCZ [69], findings from this study appear also of interest to SCZ. The researchers firstly re-analyzed a previously published meQTL dataset (see above section) from 153 cerebellum samples from BIP and control individuals [63] by inclusion of imputed genotype data and identified 5974 distinct genes associated with a *cis-*meQTL. Thereafter, they tested the hypothesis that top-ranking risk variants from GWAS for BIP are enriched for variants that affect methylation levels. In support of this hypothesis, 132 *cis*-meQTLs matching this criterion were identified. These disease-associated meQTLs compromised ≈14% of the most significant associations from two GWAS for BIP. Further, 77 of the meQTL SNPs also associated with a *cis*-eQTL indicating that BIP risk variants were enriched in both *cis-*meQTLs and *cis*-eQTLs. Consistent with previous studies, few combined QTLs were detected indicating that individual SNPs that control both DNA methylation and expression of the same proximal gene appear to be a minor fraction among the most significant associations with BIP. Among these, *DLG5*, encoding the polarity protein discs large homolog 5, appears of particular interest in view of its role in the establishment and maintenance of epithelial cell polarity. DLG5 belongs to a family of molecular scaffolding proteins called membrane associated guanylate kinases (MAGUKS) and has evolved in the same manner as DLG1 and zona occludens (ZO1) [70]. Together, these scaffolding proteins regulate cell migration and adhesion, precursor cell division and proliferation, epithelial cell polarity maintenance, and transmission of extracellular signal to the membrane and cytoskeleton. All of these processes play an important role in early neural stem cells [71] and their dysregulation could contribute to the neurodevelopmental origin of SCZ.

In summary, this study raises the intriguing possibility that many *cis*-meQTL effects on gene expression in healthy and/or diseased brains operate in a highly temporal-spatially constrained and context-dependent manner (see Figure 1).

Subsequently, detailed meQTL analysis of 110 non-psychiatric and 106 SCZ dorsolateral prefrontal cortices on the Illumina 27K array identified 36,366 *cis*-meQTLs (SNP-CpG pairs), independent of case-control status [72].

Extending these insights further, two recent landmark studies on SCZ have comprehensively explored the role of CpG methylation and of meQTLs in human fetal brain and adult brains with respect to schizophrenia risk [51,52].

**Figure 3:**
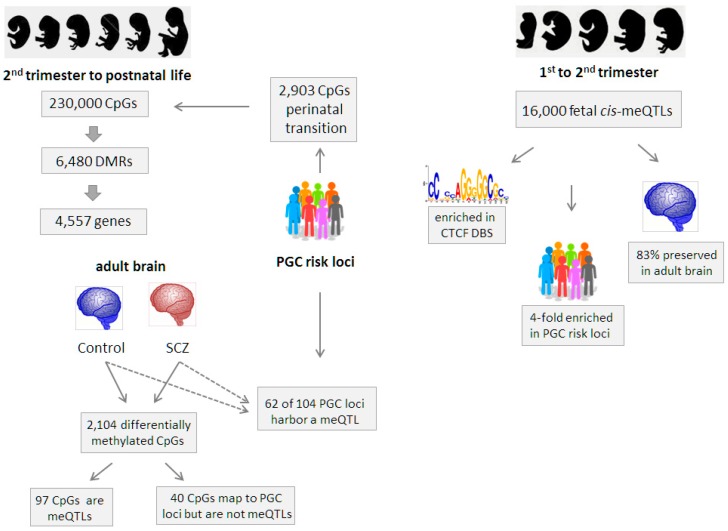
Findings on CpG methylation and on meQTLs in fetal and adult brains from control and SCZ individuals. Results from Jaffe el al. [52] (**left**) and from Hannon et al. [51] (**right**) are schematically summarized. In adult brain 62 of 104 Psychiatric Genomics Consortium (PGC) loci harbor a meQTL independent of case control status and fetal meQTLs are four-fold enriched in PGC risk loci. CTCF: CCCTC-binding factor; DBS: DNA binding site.

Jaffe and co-investigators analyzed genome-wide DNA methylation profiles in dorsolateral prefrontal cortex from 335 non-psychiatric individuals covering a wide age range from 14th week of gestation to 80 years of age. Using a two-stage analysis strategy, the authors first sought to pinpoint DMRs that arise during the transition phase from the second fetal trimester to postnatal life, independent of case control status. This analysis identified 230,000 of 456,000 autosomal CpGs to be differentially methylated corresponding to 6480 statistically significant DMRs. These DMR localized to 4557 unique genes, many with a function in brain development and morphogenesis (Figure 3, left part).

In a similar approach, Hannon and coworkers [51] investigated in a first step 166 fetal brain homogenates ranging from 56 to 166 days post-conception. Genome-wide high-density CpG methylation profiling (Illumina 450K) combined with genome-wide SNP genotyping of the same samples led to the identification of ≈16,000 *cis*-meQTLs within 1 Mb (Figure 3, right).

However, it is important to note that the effect sizes of these meQTLs are small, with a median change in DNA methylation per allele across all detected *cis*-meQTLs of ≈7% for each meQTL SNP and thus slightly higher than in a previous report (median effect size ≈4.1%) [50]. Similarly, there were only few *trans*-meQTLs (≈5%) of smaller effect size that showed a higher proportion of DNA methylation changes per allele.

These fetal brain meQTLs were enriched in regulatory domains, including DNase I hypersensitive sites, repressive histone marks, TF binding sites, and significantly overlapped with eQTLs, all of these findings are in accord with previous studies [60,67]. Interestingly, fetal brain meQTLs strongly associated with DNA-binding sites for the architectural zinc-finger protein CTCF corroborating a previous study of heritable DNA methylation sites in the human brain [73]. Functionally, CTCF is thought to coordinate the interplay between higher-order chromatin structure and lineage-specific gene expression. In line with previous findings on the methylation-sensitive binding of this zinc-finger protein, a recent study showed altered 3D chromatin structure due to hypermethylation at CTCF binding sites [74], ultimately inducing aberrant gene expression. These findings point to a potential mechanism of action underlying meQTL function and molecular consequences. In support of a role in SCZ, CTCF has been recently identified in an integrated pathway approach for pathways and genes affected in SCZ [75]. Together, these findings suggest a regulatory role for fetal meQTLs and highlight with CTCF an important mechanism connecting genomic variation to genomic function.

Going beyond fetal brain meQTLs, Hannon and Jaffe and co-workers [51,52] next sought to pinpoint potential meQTLs and differentially methylated CpGs independent of genetic changes that are associated with SCZ.

Interestingly, Hannon and co-workers could show that 2903 CpGs residing in PGC risk loci for SCZ were more likely to be differentially methylated during the transition of prenatal to postnatal life than non-SCZ risk loci (Figure 3, left). In addition, Hannon et al. detected fourfold enrichment for genome-wide significant PGC risk variants among fetal brain meQTLs, providing further support for a developmental role of SCZ associated genetic variation (Figure 3, right). In line with this hypothesis, PGC risk variants are enriched in G-protein coupled receptor signaling, glutamatergic neurotransmission, neuronal calcium signaling, synaptic function and plasticity, neuronal ion channels and several neurodevelopmental genes. Further, 83% of the fetal meQTLs were conserved in at least one of three tested adult brain regions (prefrontal cortex, cerebellum, and striatum) suggesting a potential role in later life (Figure 3, right).

Independently, Jaffe and coworkers carried out meQTL analysis on an adult cortex sample set (191 adult SCZ patients vs. 240 non-psychiatric controls) and were able to show that 62 out of 104 genome-wide significant PGC loci harbor a meQTL within 20 kb of tag SNPs and those in LD (*R^2^* > 0.6). However, it is important to note that none of these PGC meQTLs seemed to be specific to control or disease status. Nevertheless, DNA methylation levels proximal to PGC risk variants may still contribute to SCZ onset and progression by the mediation of environmental clues (Figure 3, left).

In an orthogonal line of investigation, the authors pinpointed 2104 differentially methylated CpGs that were significantly differentially methylated between SCZ cases and controls using adult brain samples. These disease status-related CpGs were slightly, but significantly enriched with SCZ risk loci (40 CpGs of 2,104 CpGs) but none of them were meQTLs to any SCZ risk SNP. At the same time, only 97 diagnosis-related CpGs fulfilled the criterion of a genome-wide significant meQTL. Conclusively, these results indicate that diagnosis-associated CpGs are not related to meQTL associated with SCZ risk loci (Figure 3, left).

In sum, these findings suggest that a major proportion of SCZ risk loci contain a meQTL (62 among 104 PGC risk loci) [52] and that fetal meQTLs, which are about fourfold enriched for PGC risk loci, largely persist (about 83%) in the adult brain [51].

## 11. Conclusions and Outlook

Accumulating evidence shows that DNA methylation is influenced by genetic variation and that abundant brain meQTLs offer a potential mechanism to couple genetic variation to complex psychiatric disorders. The high incidence of SCZ risk variants containing a meQTL and the significant enrichment of risk variants in fetal brain meQTLs together suggest that common genetic variants conferring risk for SCZ may link to altered DNA methylation in the fetal human brain and support the neurodevelopmental origin of SCZ. At the same time, disease-specific fetal meQTLs remain still to be identified.

Current research on the role of the methylome in human mental health and disease is still in its infancy and needs to face challenges from tissue heterogeneity, spatial and temporal effects in genetic variation, and ultimately functional causality. To elucidate disease processes in SCZ and to develop new therapies, it will be important to correlate fetal brain meQTLs to molecular and cellular functions. SCZ-associated SNPs associated with rather subtle changes in methylation differences accounting for 1.3% average methylation differences in diseased and control prefrontal cortex [52] and for 6.7% difference per allele for the average meQTLs [51]. Do such subtle changes in CpG methylation influence gene expression levels? The present studies did not resolve this issue and several potential caveats come to mind. First of all, both studies generated averages across large numbers of cells by using homogenized tissues that are very likely to dilute cell-type specific signals from heterogeneous brain tissues and biologically relevant variability at the level of individual cells. This limitation applies to changes in both CpG methylation and mRNA levels. Cellular heterogeneity presents a major confounding factor and statistical methods that correct for differences in cell composition can help to overcome these limitations [76,77].

Alternatively, innovative single-cell assays are now available for genome, epigenome, and transcriptome analysis [78,79] and provide promising tools to enhance cellular resolution in diseased brains. Moreover, subtle changes in CpG methylation may not drive changes in mRNA levels but cell type-specific alternative splicing [67]. In this regard, a recent report identified a large number of splicing eQTLs in Yoruba LCLs that showed effects of similar or even larger size than eQTLs [28]. This study highlights RNA splicing as a primary link between genetic variation and disease and further studies on the role of brain meQTLs in RNA splicing and SCZ are sought.

meQTLs are enriched in regulatory domains and both positive and negative correlations with gene expression have been observed. A possibility for the positive correlation between methylation levels and the expression of nearby genes are methylation-sensitive DNA-binding sites recognized by repressor proteins. Another possibility is the presence of 5-hydroxymethylcytosine (5hmC) that is generated by active demethylation and is most abundant during neurogenesis. This oxidation product maps preferentially within distal regulatory elements and gene bodies of activated neuronal function-related genes. 5hmC has activating effects on transcription [80] and possibly on RNA splicing as well [81,82,83]. Fetal brain meQTLs coincide with remodeling of the neural methylome that make it important to discriminate between different forms of CpG methylation when referring to their effects on gene transcription.

Overall, the impact of meQTLs on human brain function is still under-studied and even less understood for the pathogenesis of SCZ. Conceptually, meQTL studies map genome-wide DNA methylation levels to genetic variation but do not per se identify causal variants; a caveat that likewise applies to conventional GWAS. Hence, overlap of meQTL and GWAS finding may be biased by LD structure, and not the same causal variant influencing both DNA methylation and SCZ. To further refine SCZ candidate regions, Hannon and coworkers therefore undertook a Bayesian colocalization analysis [84] to identify variants associated with both DNA methylation and SCZ. By comparing the pattern of association results from PGC risk variants and meQTL analysis across a region they identified 65 regions supportive of a colocalized association signal for both SCZ and DNA methylation in the respective region. Among these regions was the *AS3MT* locus, a top-ranked candidate from the previous PGC study [16]. This result indicates that meQTLs can be used to localize putative causal loci within large genomic regions associated with SCZ.

While refined biostatistical methods can help to identify potential causal loci in SCZ, we still face a tremendous lack of functional studies assessing the origin and consequences of fetal meQTLs. Specifically, SCZ onset in adolescence and young adults is a major obstacle for the identification of SCZ-specific meQTLs at stages when future health status is still unknown.

In this respect, induced pluripotent stem cells (iPSCs) [85] from adult SCZ and control subjects are a promising opportunity to identify and to explore the role of fetal meQTLs since they recapitulate hallmarks of fetal brain cell types and can be differentiated into various cell types [86]. Further, forebrain-specific organoids derived from 3D culture of iPSCs show gene expression profiles remarkably similar to those of fetal tissues, and organize into cerebral cortex-like regions indicating that the genetic features underlying human cortical development can be studied in this system [87]. Progress on 3D systems is likely to enable the generation of organoids from additional brain regions of interest in SCZ and to pave the way to large-scale analysis of patient-derived iPSCs [88,89].

To assess the consequences of non-coding variants of fetal meQTLs on epigenetic modifications and gene expression in a neurodevelopmental context, select genetic variants can be engineered into isogenic human iPSCs using programmable nucleases (zinc-finger nucleases (ZFNs), transcription activator-like effector nucleases (TALENs), and RNA-guided engineered nucleases (RGENs) derived from the bacterial clustered regularly interspaced short palindromic repeat (CRISPR)-Cas (CRISPR-associated) system). These tools facilitate targeted genetic modifications in patient- or control-derived iPSCs through their ability to induce site-specific DNA cleavage in the genome, the repair of which (through endogenous mechanisms) allows high-precision genome editing.

Collectively, disease-relevant tissues derived from iPSCs provide a unique opportunity to dissect potential regulatory effects of fetal, potentially disease-specific, meQTLs in the context of spatial and temporal aspects of gene expression important to mechanisms in SCZ.

Even in well-studied tissues and cell types currently available functional annotations are incomplete and correspond mostly to non-physiological conditions. Therefore, future studies are necessary to map QTLs in human cells exposed to physiological relevant stimuli for capture of the relevant biology. For example, stimulus-dependent eQTLs in whole blood can help to explain individual differences in the transcriptional response to stress and show an enrichment in loci from GWAS for SCZ and MDD [90].

Dynamic changes in gene expression and DNA methylation are known to regulate protein synthesis and synaptic connectivity important to learning-induced neuronal activity [91]. In contrast, sustained changes in gene expression and DNA methylation could compromise neuronal plasticity by weakening the ability of neurons to respond to later stimuli. On the other hand, sustained changes in DNA methylation without changes in gene expression may encode gene expression potential that requires renewed neuronal reactivation to manifest [92]. Such a form of latent responsivity could contribute to the low correlation between meQTLs and gene expression reported so far, particularly in resting neurons. In this regard, iPSCs offer the possibility to explore the role of stimulus-dependent meQTLs during early stages of fetal development. Dynamic methylation changes during fetal and perinatal brain development presumably reflect concerted effects from genetic and environmental variables [39,93], the latter including maternal stress and infections, obstetric complications, and maternal nutrition during pregnancy; all of these have been associated with SCZ [94]. meQTLs that map to GWAS loci raise the intriguing possibility that these genomic regions encode sensitivity towards the environment by regulating DNA methylation levels. Exposure of iPSCs to stimuli that mimic environmental exposures can help to capture a potential role of meQTLs in the genes environment dialog.

A similarly attractive perspective opens when the findings of fetal epigenomics and GWAS for SCZ are considered in the context of cell type-specific epigenomics in mouse brains [95]. Different subtypes of neocortical neurons showed highly distinctive epigenomic landscapes, differing in chromatin accessibility and DNA methylation signatures characteristic of gene regulatory regions. Neuronal epigenomes encoded both past and present gene expression, with DNA hypermethylation at developmentally critical genes appearing as a novel epigenomic signature in mature neurons. This raises the exciting possibility that the neuronal epigenomes from control and disease brains preserve a trace of the expression pattern during prenatal and early development. Seemingly, integrated comparison of brain methylomes could help to define the time window during which epigenomic dysregulation arises and how this intersects with any harmful exposures of mothers and unborn children. If that is the case, information from the past could benefit timely future preventions in high risk families for SCZ.
